# Small-Group Learning in an Upper-Level University Biology Class Enhances Academic Performance and Student Attitudes Toward Group Work

**DOI:** 10.1371/journal.pone.0015821

**Published:** 2010-12-29

**Authors:** Andrew D. Gaudet, Leanne M. Ramer, Joanne Nakonechny, Jacquelyn J. Cragg, Matt S. Ramer

**Affiliations:** 1 Department of Zoology, University of British Columbia, Vancouver, Canada; 2 International Collaboration On Repair Discoveries, University of British Columbia, Vancouver, Canada; 3 Vancouver Coastal Health Research Institute, University of British Columbia, Vancouver, Canada; 4 Science Centre for Learning and Teaching (Skylight), University of British Columbia, Vancouver, Canada; University College London, United Kingdom

## Abstract

To improve science learning, science educators' teaching tools need to address two major criteria: teaching practice should mirror our current understanding of the learning process; and science teaching should reflect scientific practice. We designed a small-group learning (SGL) model for a fourth year university neurobiology course using these criteria and studied student achievement and attitude in five course sections encompassing the transition from individual work-based to SGL course design. All students completed daily quizzes/assignments involving analysis of scientific data and the development of scientific models. Students in individual work-based (Individualistic) sections usually worked independently on these assignments, whereas SGL students completed assignments in permanent groups of six. SGL students had significantly higher final exam grades than Individualistic students. The transition to the SGL model was marked by a notable increase in 10th percentile exam grade (Individualistic: 47.5%; Initial SGL: 60%; Refined SGL: 65%), suggesting SGL enhanced achievement among the least prepared students. We also studied student achievement on paired quizzes: quizzes were first completed individually and submitted, and then completed as a group and submitted. The group quiz grade was higher than the individual quiz grade of the highest achiever in each group over the term. All students – even term high achievers –could benefit from the SGL environment. Additionally, entrance and exit surveys demonstrated student attitudes toward SGL were more positive at the end of the Refined SGL course. We assert that SGL is uniquely-positioned to promote effective learning in the science classroom.

## Introduction

*“Coming together is a beginning. Keeping together is progress. Working together is success.”*

- Henry Ford

The ongoing transformation of science education [1], [2] is challenging many educators to explore new teaching strategies. Instructors at every level are encouraged to expand their repertoire of tools for teaching science to meet the following two essential criteria. First, teaching practice should be directed by our ever-expanding understanding of learning [3], [4]. Second, science teaching should align itself with the nature of scientific enquiry; that is, science should be taught as it is practiced [1]. One tool that can help instructors meet both of these goals is the use of small learning groups.

A growing body of evidence suggests small-group learning (SGL), defined here as the use of permanent small groups of 5–7 students, benefits undergraduate science student achievement. For example, peer instruction [5], [6], [7] and collaborative testing [5], [8] improved retention, flexible performance capacity (applying knowledge to solve novel problems), and performance on quizzes and exams. In addition, a meta-analysis indicated that SGL improved academic achievement and reduced attrition in undergraduate science, math, engineering, and technology courses [9].

SGL encourages students to become more engaged in material through discussion, debate, and the opportunity to articulate explanations to their peers [10], [11]. Such classroom practices allow students to check their understanding and construct new knowledge, through interactions with each other and with course material: we now appreciate that these are the requirements for meaningful learning [3], [4]. SGL may represent one of the most accessible methods for converting a classroom from a teacher-centered setting to an active, learner-centered environment.

SGL also models the collaborative and social nature of scientific practice [12]. When students confront scientific problems in groups, they have the opportunity to develop cognitive skills, attitudes, and behaviours essential for scientific enquiry. Further, SGL encourages student cooperation and student-faculty interaction, both crucial determinants for students' academic and personal development, and overall satisfaction with their university experience [13].

In an effort to update our teaching practice, we implemented an SGL model in a fourth-year developmental neurobiology course. The transition from an individual work-based (Individualistic) to an SGL environment was relatively straightforward (from the instructors' points of view), and made the course more enjoyable to teach. The obvious question was whether incorporating SGL in this instance enhanced student learning.

We studied the course section over five years in order to establish whether SGL incorporation affected student performance and attitude towards group learning. Our primary outcome measure was performance on final exams over five terms, comparing SGL terms with previous terms. As secondary outcomes, we examined performance on quizzes (individual and group), and student attitudes toward the group learning environment in our SGL classes. We show that our SGL model was associated with enhanced student performance on final exams, that students benefited from group quizzes, and that student attitudes toward group work improved after completing the SGL course.

## Methods

### Ethics Statement

This study was approved by the Behavioural Research Ethics Board (BREB) at the University of British Columbia (UBC). For the portion of the study on quiz grades and student attitudes, we obtained written consent from 39 of 46 students in the fall 2008 section of Developmental Neurobiology at UBC, and 36 students completed both entrance and exit surveys. In consultation with BREB, we are using final exam grades to examine the impact of SGL without written consent from students in those terms. There is no personally identifiable information in these data, nor is it possible to determine the grade of any student.

### Course overview

MSR, ADG, and/or LMR (in different combinations) co-instructed a one-semester, 3 hour per week, fourth-year elective course on developmental neurobiology over five terms (BIOL 458; 2005–2009). This course is one of two undergraduate neuroscience courses at UBC and there are no prerequisites for the course.

### Conversion to SGL

The conversion of the course from Individualistic to SGL can be described in three stages:

#### Stage 1: Individualistic (2005 and 2006)

Students usually sat and worked individually. The course was largely lecture-based, but some of the course evaluation components – including daily quizzes at the start of class, seminars, and in-class assignments – facilitated active learning. Two factors encountered in the course encouraged the instructors to re-think their teaching approach. The first factor was the amount of substantial misconceptions persisting for some students. Even when a section of the material was discussed at length after a quiz, it was apparent this whole-class discussion did not promote conceptual understanding for all students, as significant misconceptions were revealed in student answers on the final exam. Second, when students were given the opportunity to work on a quiz in pairs (several times throughout both terms, on particularly challenging quizzes), the atmosphere in the classroom changed dramatically, from solemn and serious, to spirited and engaged. Based on these positive collaborative experiences, we designed a SGL model to stimulate student interaction, while retaining the active learning elements that were already part of the curriculum.

#### Stage 2: Initial SGL (2007)

We devised a system to divide students “randomly” into diverse learning groups [14]. The instructors split the students into groups of six in-class (on the second day) to be transparent about the process. Instructors displayed a list of five subject majors, and asked students to self-identify with one major that best-described their area of academic expertise. Instructors then called out each subject major in turn; when their subject major was called, students were asked to form a line around the classroom (e.g. the first 20 students in the line are Cell Biology majors; the next 16 students in the line are Non-Biology majors, etc.). Students then sequentially assigned themselves a number up to 17 (there were 17 groups in 2007) and repeated this process until all students had an assigned number. Finally, all the same numbers formed a group of six. Student were required to sit with their groups immediately and for the remainder of the term. The process of group formation ensured that groups would be diverse according to subject major, and the entire process consumed approximately fifteen minutes of class time.

Students sat and worked in their permanent four- to six-person small groups. All groups initially had six students, but a few students assigned to groups withdrew from the course within the first two weeks. Most groups comprised five or six students, and we required that groups had a minimum of four members. Periods of lecture were interrupted by formative group activities at ∼15-minute intervals. We created group activities that challenged students to employ a scientific approach to analysis and evaluation of data from primary literature: groups were asked to interpret figures, to create models based on pertinent data, and to design experiments that would extend or clarify the findings in a data set [1], [15]. Seminars and in-class assignments were usually completed as a group; however, for assignments, students occasionally worked individually to reflect or review. For example, after a class on programmed cell death, individuals were asked to write a paragraph to explain the process to a relative with no science background.

**Figure 1:**
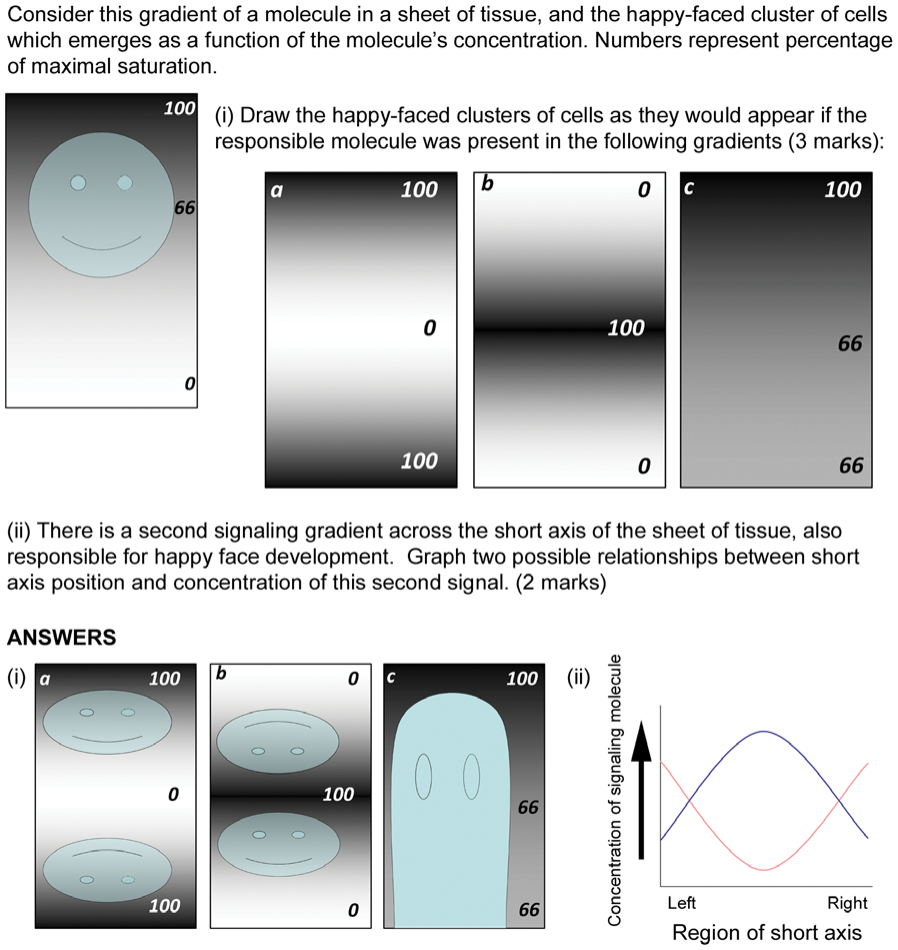
Example of a quiz question used in class. This question was designed to test the conceptual understanding of gradients of signaling molecules. Although no course-specific content is required to answer this question, most quiz questions (and those on other evaluations) required the students to demonstrate both knowledge of content and an understanding of fundamental concepts. Students were awarded part-marks for answers that were partially correct, at the instructors' discretion. For example, if a student drew only one happy face in i) a or i) b, but it was shown in the correct orientation, the student received 0.5 out of 1 for that question.

Daily quizzes were either completed individually (“individual”) or by each group as a whole, with one paper turned in per group (“group”) (see Figure 1 for a sample quiz question). Quizzes were designated as “individual” or “group” by coin toss or lottery. Quizzes were held at the end of each class to encourage student preparation and immediate active review.

From the perspective of the instructors (ADG and LMR), SGL had a dramatic positive impact: students appeared to engage more deeply with the material, and classroom discussions were lively and frequent. However, some students reported anxiety associated with the quizzes. Some students could not participate confidently in group discussions because they did not have time to read the quiz questions before a group member jumped in with an answer. Additionally, many students found it stressful to be tested on new material on the same day.

#### Stage 3: Refined SGL (2008 and 2009)

**Figure 2:**
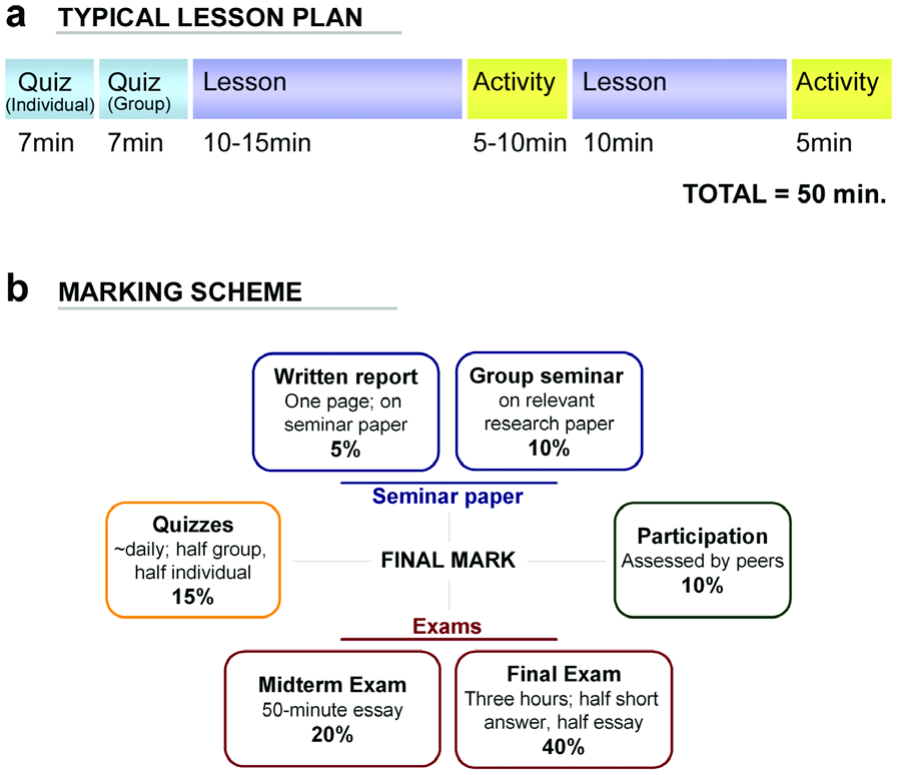
Typical lesson plan and marking scheme used in our fourth-year undergraduate biology course. a) Typical lesson plan used in the Refined SGL sections. Lessons began with a quiz on the previous day's material: this quiz was completed by individuals first, and the same quiz was then attempted by the group. The quiz was subsequently taken up in-class. The rest of the lesson consisted of instructor-led lecture and discussion, interspersed with student-centred activities. Some lessons (6 per term) were entirely devoted to in-class group assignments; other lessons (8 per term) were devoted to seminar presentations. b) Marking scheme used in the Refined SGL sections. Students were evaluated using a variety of instruments. Group-based evaluations included the group seminar, group quizzes and assignments, and participation, whereas individuals were evaluated on their written report, individual quizzes, and midterm and final exams. In order to reinforce the importance of learning concepts rather than memorization, we allowed students one double-sided “cheat sheet” for both exams. Participation marks were based on peer assessment: each student rated the performance of every other member of their group according to five categories (attendance and daily participation, preparation, responsibility, respect and seminar participation).

Students sat and worked in permanent small groups, and the course design was largely similar to 2007, except for the quizzes. Based on feedback from 2007, quizzes were held at the beginning of class and covered material from the previous day. Rather than alternating between individual and group quizzes, each quiz was first completed individually and submitted, and then the same quiz was completed in groups (Figure 2a). This format allowed all students to consider each question alone and commit to an answer before tackling the question in their groups. Individual and group quizzes each accounted for 7.5% of the final course grade (Figure 2b).

### SGL effects on academic performance

**Figure 3:**
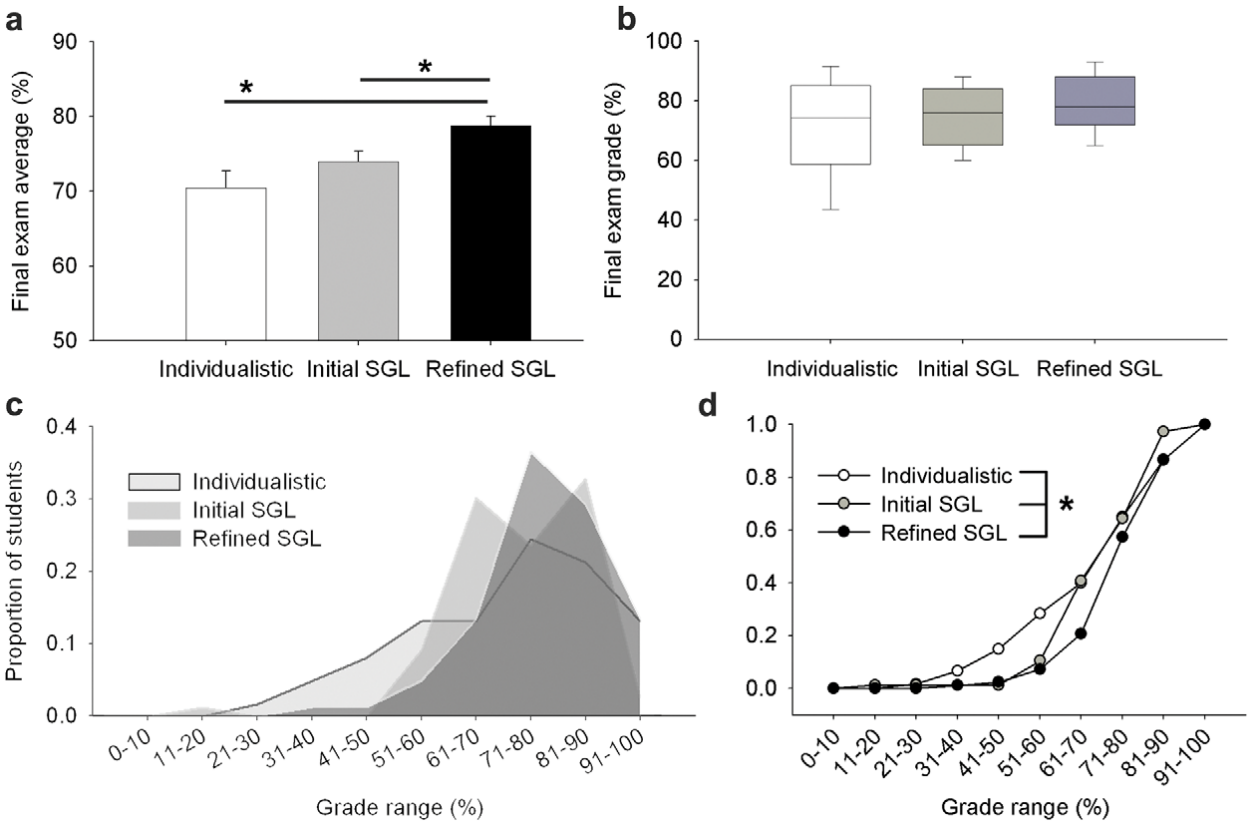
The effectiveness of small-group-learning (SGL) is borne out in final exam performance. Grades from final exams were examined for five terms of a fourth-year Developmental Neurobiology course. Terms with similar learning environments were combined: students either worked in an Individualistic environment (2005 and 2006) or worked in permanent small groups (Initial SGL, 2007; and Refined SGL, 2008 and 2009). We incorporated changes in Refined SGL that improved on the design of the Initial SGL setting (e.g. by introducing individual quizzes prior to group quizzes). a) The class average for the final exam was higher in the Refined SGL environment than it was for both other groups. b) Box plots of the same data reveal an increase in median (line), 75^th^ percentile (upper limit of box) and 25^th^ percentile (lower limit of box) with the introduction of SGL. Whiskers represent the 10^th^ and 90^th^ percentiles: the grade at the 10^th^ percentile exhibits a remarkable, positive shift with the introduction of SGL. c,d). Comparison of exam grade distributions between these three groups showed that students in the Initial SGL performed better than students in the Individualistic setting, and that students in the Refined SGL environment performed better than students in both other groups. This indicates that our SGL environment benefits academic performance, and that refinements made since its initial implementation have made the SGL model more effective. Asterisks indicate significant differences between groups (Kruskal-Wallis ANOVA, Dunn's test (a); Kolmogorov-Smirnov goodness-of-fit test (d)); n = 61 (Individualistic), n = 76 (Initial SGL), n = 82 (Refined SGL).

In order to assess the effects of SGL on academic performance (Figure 3), we examined grades from the final exam for five terms of the course. We used the final exam as an outcome measure for three main reasons: first, it was used consistently in the same format over all five terms; second, it is a measure of *individual* academic performance (versus the overall course mark, which incorporates both individual and group marks); and third, it is administered at the end of the term when the effects of SGL would be most apparent. The essay questions on every exam were similar in scope and difficulty, and some essay questions were used in multiple terms. Students in terms with similar learning environments were combined: i.e., we compared exam performance between students who did not work in permanent small groups (Individualistic; 2005 and 2006; n = 61; both taught by MSR); students who worked in the Initial SGL environment (2007; n = 76; taught by LMR and ADG); and students who worked in the Refined SGL environment (2008 and 2009; n = 82; both taught by ADG and MSR). In sections taught by more than one instructor, five essays were graded independently and compared to ensure consistency in criteria and grades awarded before marking the remaining essays.

**Figure 4:**
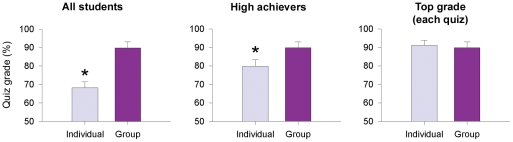
Small-group learning benefited all students, including term high achievers. In the Refined SGL environment, students first wrote each quiz individually, and then completed the same quiz in small groups immediately after (13 quizzes over the term). For the group quiz, group members had to consider and debate possible answers, since a single answer was submitted and graded for the entire group. When all quiz grades were considered, group quiz grades were significantly higher than individual quiz grades (left panel). Interestingly, group grades were also significantly higher than individual grades of term high achievers (middle panel). Term high achievers were those students who had the best average individual quiz grade over the term in each group (one student per group). Average group quiz grades were not significantly different from the individual grades of the daily top-performing student on each quiz (right panel). These data suggest that even term high-achieving students profit from small-group learning, that groups perform as well as their daily top-performing student on any given quiz, and that the daily top performer in groups changes from quiz to quiz. Asterisks indicate p<0.05 versus group quiz grades (paired t test); n = 39 (all students), n = 8 (group grades, term high achievers, daily top performer).

As an adjunct measurement of student performance in the Refined SGL environment, we examined quiz performance in 2008 (Figure 4). Since the same quizzes were completed individually and as a group (13 quizzes), we could compare individual and group quiz performance. We defined two cohorts of students based on individual quiz grades. “Term high achievers” were those students with the best average grade on individual quizzes *over the term*. We also examined grades of the “daily top performers”, those students who achieved the highest individual quiz grade *on any given day*.

### Student attitudes toward SGL

As a final effectiveness indicator of our Refined SGL environment, we used the Student Attitudes toward Group Environments (SAGE) questionnaire [16] to determine how student attitudes toward group learning changed over the term in 2008. For this portion of the study, students were recruited during the first week of class by JJC or LMR, when she was not instructing). Students were informed that: 1) participation was entirely optional and would not affect their grade in the course; and 2) the instructors would not be aware of who chose to participate until final grades for the course were submitted. Surveys were distributed and completed during class time.

To complete the SAGE questionnaire, students chose their score on a five-point Likert scale for each of 54 “attitude statements” (see [16] for the questionnaire – we adhered to their methods for analysis). Each of the items in the questionnaire contributed to one of four subscores:

- Quality of Product: relates to the perceived academic benefits of working in groups; e.g. “When I work in a group, I do better quality work.”
- Peer Support: measures the extent to which students feel valued within the group; e.g. “I feel I am part of what is going on in the group.”
- Student Interdependence: measures how equal and important students feel each others' contribution is to group product; e.g. “Everyone's ideas are going to be needed if we are going to be successful.”
- Frustrations: relates to feelings surrounding challenges commonly associated with group work; e.g. “I become frustrated when my group members do not understand the material.”

In addition to the SAGE questionnaire, students were asked to respond to two short-answer questions as part of the entrance and exit survey. We asked: “What is/was positive, beneficial, or valuable about working in a group environment?” and “What limitations have you/did you encounter while working in a group?”.

### Data analysis and statistics

Data were compiled and analyzed using SigmaPlot 2001 (SPSS, Chicago, IL). Statistics were performed using SigmaStat 3.0 (SPSS). Average exam grades were compared across groups using a Kruskal-Wallis One Way Analysis of Variance (ANOVA) on Ranks; Dunn's Method for pairwise multiple comparisons was used to detect inter-group differences. Quiz scores and student attitudes were compared using paired Student's *t*-tests or their non-parametric equivalent (Wilcoxon Signed Rank Test). Data are presented as mean ± standard error of the mean. Differences were considered significant at *p*<0.05.

The Kolmogorov-Smirnov goodness-of-fit test was used to determine whether exam grade distributions differed significantly. In this test, a D-statistic is calculated from the maximum difference between the cumulative distribution plots of two data sets. It does not produce a P-value as output; instead, the D-statistic is compared with a critical value. For samples >35, the critical value at the 0.05 level is 1.36/(n)^0.5^, where n =  sample size. If the critical value is greater than the D-statistic, the distributions are not signficantly different.

## Results

### Effects of small-group learning on student achievement

In order to determine whether incorporating SGL improved academic performance, we examined final exam grades for five course terms (Figure 3). The exams, written individually, consisted of essay questions that were broad in scope, and required students to synthesize material from many different parts of the course. For example, one question was, “How do concentration gradients participate in the development of the nervous system? Consider effects on cell fate and on axonal guidance.” The essay questions on every exam were similar in scope and difficulty. Results from terms with similar learning environments were combined: we compared exam performance between students who did not work in permanent small groups (Individualistic; n = 61); students who worked in the Initial SGL environment (n = 76); and students who worked in the Refined SGL environment (n = 82).

In the Refined SGL environment, the class average on the final exam was increased relative to both Individualistic and Initial SGL environments (Individualistic: 70±2%; Initial SGL: 74±1%; Refined SGL: 79±1%) (Figure 3a). Box plots of the same data (Figure 3b) reveal a higher median (line), 75th percentile (upper limit of box) and 25th percentile (lower limit of box) with the introduction of SGL. Whiskers represent the 10th and 90th percentiles. Interestingly, the grade at the 10th percentile was dramatically increased after the introduction of SGL. The 10th percentile on the final exam was at 47.5% (nearest rank) in the Individualistic setting. In contrast, the 10th percentile was increased to 60% in the Initial SGL environment, and to 65% in the Refined SGL environment. These results suggest that SGL implementation (and refinement) had a positive impact on student achievement on the final exam. Moreover, the incorporation of SGL was associated with striking improvements in the achievement of low-scoring students.

We also compared the distribution of grades on the final exam for each of the three groups (Figure 3c,d). Significant differences in grade distribution were detected using the Kolmogorov-Smirnov goodness-of-fit test. Again, we found that students in the Initial SGL environment performed better than students in the Individualistic setting, and that students in the Refined SGL environment performed better than students in both other groups. This performance indicates that our SGL environment benefits academic performance, and that refinements made since 2007 (Initial SGL) have made the SGL model more advantageous for students.

Although a wealth of data show that collaborative work enhances student achievement [17], [9], one potential concern is that high-achieving students may be limited or frustrated by the group environment. To study the effect of group work on high-achieving students' performance, we examined their grades on group and individual quizzes over the term in the Refined SGL environment (Figure 4). First. individuals completed the quizzes and handed them in without any grading occurring; then groups completed the same quizzes and one answer sheet per group was handed in. If the groups did not engage in meaningful discussion during quizzes and simply deferred to a student who generally performed best on individual quizzes, then the individual quiz grades of the term high achievers (those students with the best average individual quiz mark in each group over the term) might not be different from the group quiz grades. This effect could also occur if the quizzes were too easy. Conversely, if the groups thoroughly debated potential answers, the group quiz grades might be higher than the individual quiz grades of term high achievers, since other group members might be able to contribute ideas that lead to a higher quiz score.

Interestingly, we found even the term high achievers were out-performed by their groups on quizzes (all individuals: 68±3%; term high achievers: 80±4%; group: 90±3%). Group performance on each quiz matched the daily top-performing students, those students who achieved the highest individual quiz grade *on any given day* (daily top performers: 91±3%) (Figure 4). This performance suggests the group discussion process was successful: rather than simply deferring to their term high achiever (the student with the highest average over the term who may have gained a reputation as a reliable source of information), groups generally arrived at answers that concurred with those of their daily top-performing member. Course and quiz design seemed to encourage group discussion and debate about potential answers before writing down a single answer for the group. Therefore, in addition to the learning benefits associated with articulating explanations [10], term high-achieving students benefit from the explanations provided by their peers in this small-group setting.

### Effects of small-group learning on student attitudes

**Figure 5:**
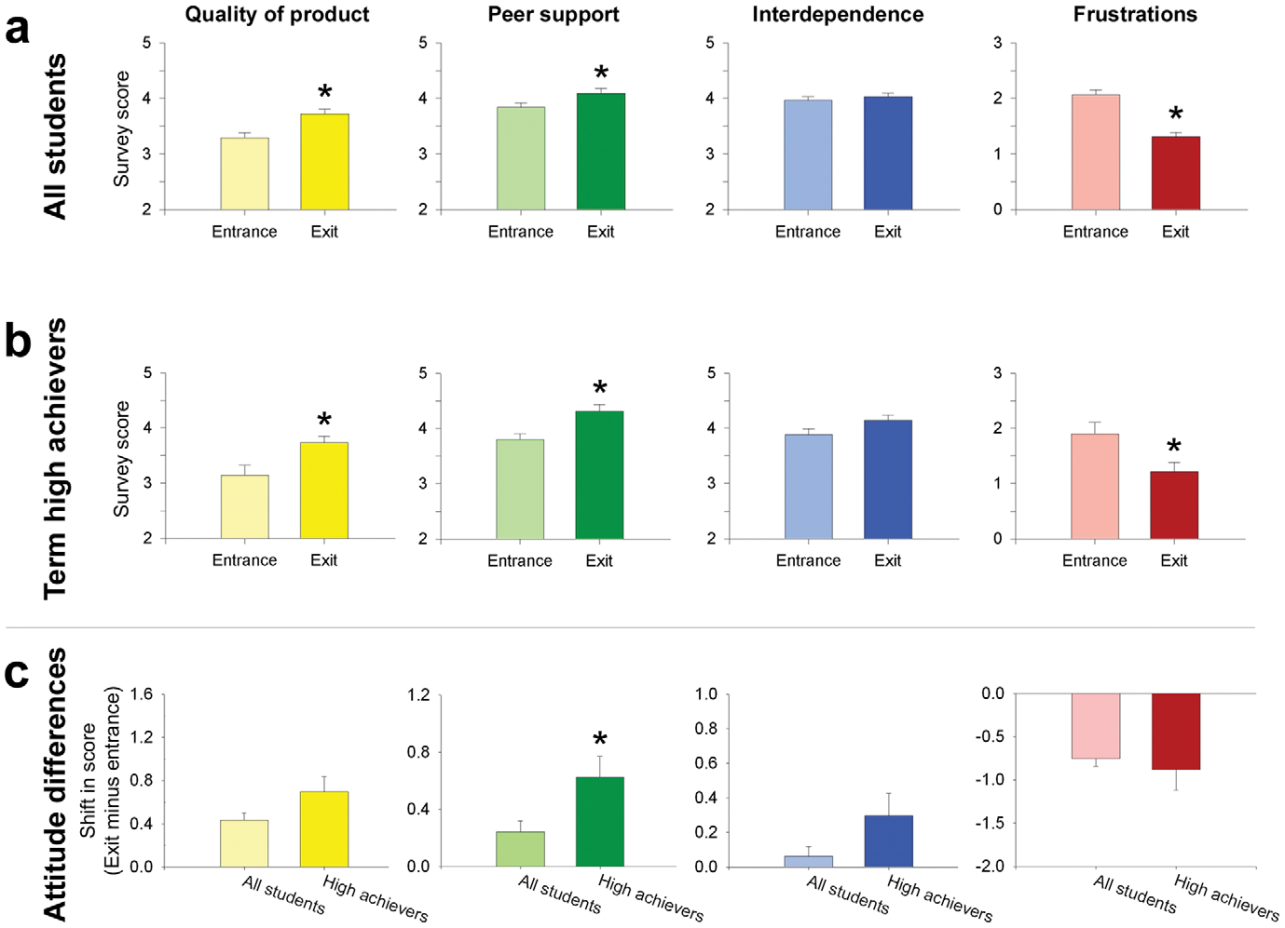
Student attitudes toward small-group learning improved over the term. Students in the Refined SGL environment completed the Student Attitudes toward Group Environments (SAGE) questionnaire twice, once at the start (Entrance) and once at the end (Exit) of the course. Each question on the SAGE survey contributed to one of four subscores. (a) Student attitudes toward the quality of product produced in groups and toward peer support offered in groups were more positive on the exit survey than on the entrance survey. There was no change in attitudes toward student interdependence. Students expressed much less frustration with group members on the exit survey. (b) The positive shifts in attitude expressed by all students were also reflected in term high achievers' responses. (c) High achievers' attitudes towards peer support shifted significantly more over the term than did the attitudes of all students. Entrance survey scores were subtracted from exit survey scores for each of the four subscores. These data suggest that students – even term high achievers – develop a more positive perspective toward working in groups after taking a course structured around SGL. Asterisks indicate p<0.05 versus entrance survey (a, b; paired t test) or versus all students (c, unpaired t test); n = 36 (all students), n = 7 (term high achievers – one term high achiever did not complete both surveys).

We were interested in how students' attitudes toward group learning might change with an SGL experience as many undergraduate science students are not accustomed to SGL-based courses. To assess student attitudes about the Refined SGL environment, we administered the SAGE survey [16]. Each SAGE Likert item contributes to one of four sub-scores (Figure 5). On three of the four subscores – Quality of Product, Peer Support, and Frustrations – the students' attitudes exhibited a significant positive shift between the start (entrance survey) and end (exit survey) of the course (Figure 5a). The shift in the Frustration subscore was particularly dramatic: a lower score on this scale reflects less frustration, and most students were much less frustrated with group work on the exit survey (Entrance: 2.1±0.9; Exit: 1.3±0.8). Only the values for the Student Interdependence subscore did not change over the term. This subscore measures the value that students place on equal contributions of group members in the SGL environment. The Student Interdependence subscores were quite high on the entrance survey, which may explain why there was no shift in the subscore on the exit survey. Importantly, when term high achievers were considered separately, we found their attitudes toward group work also improved over the term (for the same three subscores; Figure 5b). In fact, term high achievers' remarkable positive shift in attitude towards peer support over the term was significantly higher than the shift recorded from all students that completed the surveys (Figure 5c) (exit survey minus entrance survey; All students: +0.24±0.08; Term high achievers: +0.6±0.1). Overall, these quantitative data show all students had a more positive perspective about group learning after one term in the Refined SGL environment.

#### Perceived benefits of SGL

To provide students with the opportunity to discuss their SGL experience, we asked two open-ended questions: “What is/was positive, beneficial, or valuable about working in a group environment?” and “What limitations did you encounter while learning in a group?” The responses to the first question could be generally grouped into four categories: enhanced understanding, hearing different perspectives, increased motivation, and social support.

Many students identified benefits that could be described as “enhanced understanding”. For example, one student appreciated “the opportunity to teach each other – it really clarified/solidified the material.” Another student said, “Everyone can help the rest of the team understand and learn the material better since everyone has different strengths.” One student reported that working in groups helped to eradicate the illusion of comprehension [18]: “Sometimes I think I understand something until a friend asks me a question and I can't give … the EXACT answer.”

Many students also valued the opportunity to hear others' perspectives. One student's comment read, “We got the opinion of many different classmates from different backgrounds.” Another student remarked, “My insight toward the course became broader, since I got the different views [which] enabled me to think in different ways.”

Several students reported that group learning enhanced their motivation (“It keeps me on the ball” and “Work was more fun”), and allowed them to meet peers (“It is nice to meet and develop friendships”). The social importance of SGL became clear as students reported they “got to know classmates better compared to other classes”; “It is nice to meet and develop friendships”; and even that “it was nice to have someone to sit with.”

#### Perceived limitations of SGL

Students' responses to the question, “What limitations did you encounter while learning in a team?” could also be readily grouped into four categories: coordinating schedules, managing time in-class, disparate motivation, and communication.

Many students identified time management, both inside and outside the classroom, as factor that made group work difficult. A few students identified in-class time management as a limitation of group learning (“Lots of people, little time”). However, as only a minority of students cited this as a limitation, it appears most groups had ample time to arrive at a consensus on in-class assignments. Outside-class time management problems were often related to scheduling difficulties. The sentiment expressed by one student, “The group was too big because it was hard to get together to work on the presentation.” described the challenge many students encountered as they tried to schedule group meetings for their one out-of-class assignment, the group seminar.

Students were concerned about disparate motivation and balancing workloads entering our SGL-based course. On the entrance survey, many students (44%) worried that “people do not do their work/fall behind/are not reliable” when working in groups. Substantially fewer students (8%) recognized student apathy and motivational issues as limitations on the exit survey, which suggests that most students were generally satisfied with their group mates' effort and contribution toward the group product.

Approximately one-third of students (on both the entrance and exit surveys) cited difficulties about communicating ideas in the group. Comments included, “There were lots of opinions,” “Sometimes different interpretations of a question,” and “It was sometimes difficult for everyone to agree on one thing.” Such statements suggest the group learning environment created an intrinsic state of tension thought to be critical for effective group learning [16], [13].

## Discussion

We studied how the implementation of an SGL model affected student achievement and attitudes toward group work in a fourth-year neurobiology course. We found that students in the SGL classes (Initial and Refined) had significantly higher final exam grades compared to students in the Individualistic classes. SGL was associated with a substantial shift in the 10^th^ percentile on the final exam, suggesting that vulnerable students benefited significantly from the altered course format. We also studied grades from paired quizzes, and found that term high achievers were out-performed by their groups on these quizzes, implying that even these high-achieving students could benefit from group discussion. Finally, we found that student attitudes toward group work were generally more positive at the end than at the start of the term. In summary, our SGL model was associated with learning benefits for high- and low-achieving students, and with positive attitude shifts toward group work.

### SGL enhances student academic achievement

We showed that both low- and high-achieving students have the opportunity to increase their learning in the SGL setting. The changes in the 10^th^ percentile final exam grades after SGL implementation were particularly striking. Although most of the changes to course structure with SGL involved the incorporation of more group learning, the evaluation of student achievement in this case was an individually-written essay on the final exam. In order to perform well on this essay students had to synthesize various concepts into a coherent piece of work. The 10^th^ percentile shifted 17.5% with SGL incorporation (Individualistic *vs*. Refined SGL), suggesting that the SGL environment enabled low-achieving students to develop a better conceptual understanding of the material over the term. It is particularly noteworthy that the SGL environment produced such significant benefits for individual performance.

Our data are congruent with findings from meta-analyses, which showed SGL has robust effects on student achievement. Johnson et al. [19] compiled results from 305 studies on cooperative, competitive, and individualistic learning since 1924, and found that cooperative techniques were substantially better at promoting learning compared to the other two approaches (also see [13]). Springer and colleagues [9] integrated information from 39 studies since 1980 on undergraduate students in science, mathematics, engineering, and technology, and found a strong positive relationship between SGL use and academic achievement.

Some recent data suggest that low-achieving students, in particular, profit from learning in a group setting. Carini et al. [20] studied a population of 1058 students at 14 U.S. colleges and universities, and found that the students in the lowest quintile on standardized tests (RAND, GRE) reaped the largest benefit from the use of “active and collaborative learning” in the classroom. In contrast, active and collaborative learning were not associated with significant changes in the grades of students in the top quintile. Changes in the grades of low-achieving students are likely to be more conspicuous, since there are more grades still available to them compared to high achievers, who have relatively little room for improvement academically.

Importantly, our findings indicate that high-achieving students also have the opportunity to benefit from the SGL environment. Although group grades are not a direct measure of individual student achievement, the fact that group quiz grades were significantly higher than individual grades of term high achievers suggests that even these high-achieving students can learn from group mates in SGL. A limitation of the current study is the use of group grades as a measure of academic achievement. It is not possible to conclude that students are truly learning the correct answer or reasoning through SGL simply because their group marked down the correct answer as it is possible there was little debate or thought-provoking discussion when working on group quizzes. However, instructors consistently overheard meaningful and animated group discussions during quizzes and assignments, suggesting that the SGL environment successfully promoted student-student interaction. We plan to examine group process in the SGL setting more thoroughly in future research.

There are other published data which suggest that SGL is beneficial for high-achievers. Upon review of the relevant literature, Slavin [21] reported that the majority of studies found equal benefits of cooperative learning for high, average, and low achievers in comparison to their counterparts in control groups. In addition, recent studies by Giuliodori and colleagues studied a peer instruction model of group learning (groups of two): they found that when completing quizzes in groups after completing them as individuals, students most frequently choose the correct answer, regardless of whether the higher- or lower-achiever had the answer correct on an individual quiz question [22], [23]. Our findings extend this precedent by studying a permanent group (rather than paired quiz) learning setting, and indicate that all students, high-achieving or not, are peer learners within their groups and receive performance benefits by participating in group discussion. The finding that high-achievers stand to benefit from working in groups is of great interest, since it may help instructors sell SGL to students, and address concerns surrounding the “Hitch Hiker' problem (see Student Attitudes toward SGL, below).

### SGL and conceptual understanding

The shift observed in the final essay exam grades suggest that SGL students had a better conceptual understanding of the course material than Individualistic students. Group learning has been shown to enhance critical thinking abilities. Discussion with peers can lead to the development of a new conceptual understanding, so students can answer conceptual questions better as an individual than they did prior to the group work [24]. Other studies have shown that group learning enhances scores on questions testing critical thinking [25] and improves the process of problem-solving [26]. Therefore, SGL seems to promote students' understanding of important concepts.

### Student attitudes toward SGL

Despite well-documented benefits of group learning, many students remain reluctant to participate in group work. One of the most commonly cited concerns has been described as the “Hitch Hiker Problem” [27], the concern that high-achieving students will be limited or frustrated by working with peers who are not their equals in aptitude, experience, or motivation. In keeping with other researchers' findings [27], our data indicate term high-achieving students were not frustrated working within an SGL environment. Using the SAGE questionnaire, we found all students, including term high achievers, exhibited a positive shift in attitude as a result of their course experience (Figure 4). It is particularly telling that term high achievers reported being less frustrated with group work as a result of their course experience. This response suggests that term high achievers, like science educators, may have concerns about an SGL environment that are allayed by a successful experience with SGL.

### Key aspects of our small-group learning environment

Our small-group environment was designed to promote cooperation, rather than competition, to facilitate trust for working together to learn. In order to achieve this, we carefully considered three aspects of our model: the composition of the groups, the nature of group activities, and accountability in evaluation. We did not examine the impact of any of these aspects of SGL in this study: we mention them here because the available data suggests that they are critical to the success of SGL. Thus, we believe they are important factors for instructors considering the use of SGL in their classroom.

#### i) Group composition and formation

Most data support instructor-formed groups (cf. [28]) and any form of group diversity, including academic ability, class, gender, and ethnicity, increases cognitive disequilibrium that helps achieve deeper learning [17]. We created diverse groups of five to six students [14] that were based on subject major. This approach allowed students to self-identify with a major and facilitated non-threatening transparent group formation. Instructors further facilitated group identity construction by requiring each group to choose a nickname at the beginning of the course.

The permanency of the groups was an important element of our group design. We required students to sit together throughout the term, and to work together on group assignments/quizzes inside and outside of class. Group permanency allowed groups to develop into cohesive, efficient units, and it also contributes to students feeling a sense of responsibility to their group [14]. Meta-analysis data indicate that greater total time spent in groups has more favourable effects on undergraduates' attitudes [17], [9].

#### ii) Nature of group activities

Group work is most valuable when students are in the zone of proximal development [8] where they are challenged, but not overwhelmed, in their learning. We designed the quizzes and assignments to ensure group cooperation would facilitate learning [29]. We used a variety of evaluations – written, oral, and visual or conceptual questions and solutions (e.g., Figure 1) – so that group members had the opportunity to draw on each others' perspectives and abilities. Whenever possible in-class activities and quizzes consisted of complex questions with simple answers (e.g. multiple-choice). This type of question construction facilitated group consensus, ensured groups could provide their answers simultaneously, and made evaluation easier [14]. Simultaneous responses allowed the instructors to quickly gauge student understanding in a mid-lesson activity, and also facilitated discussion as we could ask a group how they arrived at their answer, or whether they were debating between two answers.

#### iii) Assuring accountability

Incorporating personal responsibility into evaluation is widely-recognized as a critical component for successful group learning implementation [13]. We accomplished this by requiring students to complete quizzes individually before attempting them as a group, and by including group member peer evaluation in students' final marks (10% of the final grade) (Figure 2). Students were provided with the peer evaluation marking scheme on the first day of class.

### Value of small-group learning in science teaching

From an instructor's perspective, the most rewarding facet of our SGL environment was the sense of camaraderie and cohesiveness in the classroom. Although performance and participation expectations were high, the vast majority of students remained positive throughout the term. As well, instructors designed assignments at appropriate levels of difficulty and focussed on cooperative rather than competitive learning. The learning environment was collegial, and group discussions provided instructors with many opportunities to interact with individual students. The benefits observed in the classroom were corroborated in the achievement and attitude findings where we found all students valued working in collaborative groups and learned more effectively.

In summary, our research indicates that SGL enhances the process of learning and discovery, part of the conceptual and factual synthesis necessary for scientific thinking. SGL is uniquely positioned to address challenges in 1) engaging students in order to promote meaningful learning, and 2) teaching science as it is practiced. Finally, the longer-term benefits of SGL, given students' positive shift in attitude, have important ramifications for scientific research. Academically, the development of scientific ideas or discoveries is relying more and more heavily on the efforts of groups or consortia. It is our hope that improved attitudes toward group learning fostered as students learn science will make for more effective professional collaboration, and ultimately, for more fruitful careers as science practitioners.
